# Etiology and therapeutics for cognitive dysfunction in multiple system atrophy

**DOI:** 10.4103/NRR.NRR-D-25-00681

**Published:** 2025-09-29

**Authors:** Yasuo Miki, Koichi Wakabayashi

**Affiliations:** Department of Neuropathology, Biomedical Research Center, Hirosaki University Graduate School of Medicine, Hirosaki, Japan

Neurodegenerative disease is characterized by the presence of inclusion bodies containing abnormal toxic proteins in the central nervous system. Physiological α-synuclein exists in the form of a monomer or dimer at the presynaptic nerve terminal. It serves as a key molecule to modulate endocytosis and exocytosis. However, under pathological conditions, α-synuclein adopts different conformations, being converted into toxic oligomers. The molecular weight of α-synuclein oligomers ranges from 25 to 180 kDa, and they do not form filamentous aggregates of α-synuclein. Subsequently, α-synuclein oligomers change to aggregates, including protofibrils and fibrils (Miki et al., 2022). This process has been implicated in the pathogenesis of neurodegenerative diseases collectively termed synucleinopathies, which include Parkinson’s disease, dementia with Lewy bodies, and multiple system atrophy (MSA).

MSA is a rare, sporadic neurodegenerative disease that manifests with Parkinsonism, cerebellar ataxia, and autonomic dysfunction. Individuals with MSA typically develop these symptoms in their late 50s, accompanied by a rapid progression of the disease. Within a timeframe of five to eight years following diagnosis, they become wheelchair-bound, with an approximate life expectancy of up to 10 years. To date, no pharmaceutical interventions have been discovered that can not only cure the disease, but also slow down the progression of the disease process. Pathologically, abnormal strains of α-synuclein accumulate within oligodendrocytes (glial cytoplasmic inclusions and glial nuclear inclusions) and neurons (neuronal cytoplasmic inclusions [NCIs] and neuronal nuclear inclusions). Neurons and oligodendrocytes undergo neurodegeneration while abnormal strains of α-synuclein accumulate within cells. MSA is now classified into two clinical subtypes, depending on the predominant motor presentation: a cerebellar variant reflecting olivopontocerebellar atrophy (MSA-C) and a parkinsonian variant based on striatonigral degeneration (MSA-P). However, α-synuclein-positive pathological inclusions (glial cytoplasmic inclusions, glial nuclear inclusions, NCIs, and neuronal nuclear inclusions) are widely distributed beyond the striatonigral and olivopontocerebellar systems with considerable variations among cases. This may lead to a broad spectrum of clinical manifestations of MSA, in addition to the triads (Parkinsonism, cerebellar ataxia, and autonomic dysfunction). Koga et al. (2015) reported that 29 (37%) of 79 patients with pathologically proven MSA developed mild to moderate cognitive dysfunction. However, the etiology of cognitive dysfunction in MSA remains to be fully elucidated.

**Figure 1:**
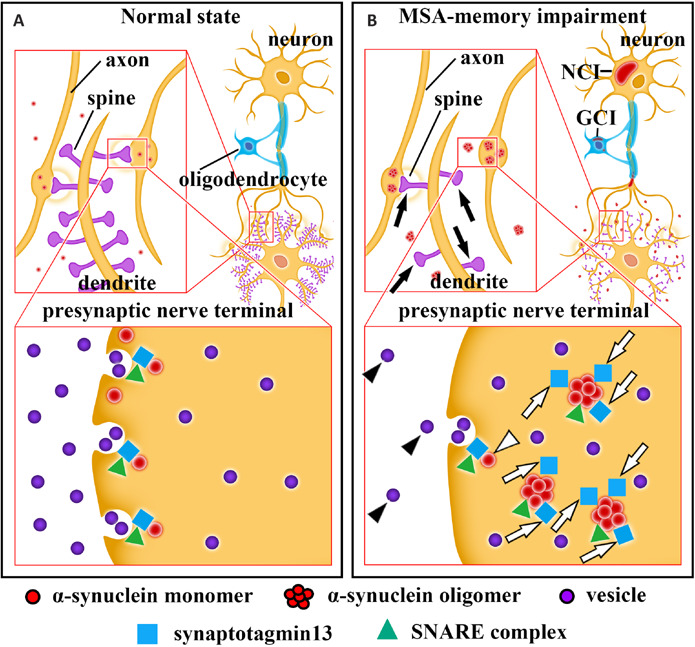
A hypothetical mechanism for memory impairment in MSA. (A) The left panel shows the normal state of the presynaptic nerve terminal in the hippocampus of a healthy individual. Physiological α-synuclein monomer (red circle) is associated with the release of extracellular vesicles, alongside synaptotagmin 13 (blue rectangle) and the soluble N-ethylmaleimide-sensitive attachment protein receptor (SNARE) complexes (green triangle). (B) In patients with MSA who develop memory impairment, toxic α-synuclein oligomers accumulate in the presynaptic nerve terminals and synaptic clefts of the hippocampus. A higher degree of interaction is observed between α-synuclein oligomers and synaptotagmin 13 (white arrows), with a decrease in the number of physiological α-synuclein at the presynaptic nerve terminal (white arrowheads). These events lead to a reduced number of dendritic spines (arrows). In addition to fewer physiological α-synucleins (white arrowheads), α-synuclein oligomers impair the release of extracellular vesicles (arrowheads) through their abnormal interaction with synaptotagmin 13 and SNARE complexes. GCI: Glial cytoplasmic inclusion; MSA: multiple system atrophy; NCI: neuronal cytoplasmic inclusion.

To address this knowledge gap, we examined the clinical records of 148 individuals with pathologically proven MSA from the archive of the Queen Square Brain Bank for Neurological Disorders between 2002 and 2018 (Miki et al., 2020). 30 (20%) of these patients had cognitive impairment, with memory impairment being present in 60% of these cases. Notably, the occurrence of memory impairment was correlated with the number of α-synuclein-positive NCIs, not glial cytoplasmic inclusions, in the hippocampus (Miki et al., 2020). To further examine the pathological substrate of memory impairment in MSA, we established a human α-synuclein-inducible mouse model of MSA, which develops memory impairment four weeks after human α-synuclein induction (Miki et al., 2022). In this model, human α-synuclein mRNA was first expressed in oligodendrocytes, and then in neurons, suggesting intercellular propagations of human α-synuclein mRNA, probably via exocytosis and endocytosis. Human α-synuclein initially accumulated in oligodendrocytes, subsequently spreading to the cytoplasm of excitatory hippocampal neurons (NCI-like aggregates) (Miki et al., 2022, 2025). Interestingly, concomitant with the formation of human α-synuclein-NCI-like aggregates, α-synuclein oligomers increased in the hippocampus. Furthermore, we found a significant decline in the number of dendritic spines, followed by a suppression of long-term potentiation (Miki et al., 2022). Consistent with data obtained from the MSA mouse model, human MSA cases with memory impairment had more α-synuclein oligomers in the hippocampus compared with those without (Miki et al., 2022). These findings suggest that the presence of α-synuclein oligomers may cause synaptic dysfunction and memory impairment in the hippocampus of MSA. Next, we tried to provide molecular insights into memory impairment in MSA. We identified ten significantly altered transcripts of vesicular transport proteins in a transcriptomic analysis using the MSA mouse model. Immunohistochemistry using human MSA cases revealed the incorporation of synaptotagmin 13 into glial cytoplasmic inclusions. Immunoblotting also showed increased protein levels of synaptotagmin 13 in the synaptosome fractions in the temporal cortex of individuals with MSA compared with those of control cases. Of note, the levels of α-synuclein oligomers paralleled with the levels of synaptotagmin 13. *In vitro* and *in vivo* analyses confirmed a decreased release of extracellular vesicles associated with abnormal interactions between synaptotagmin 13 and the soluble N-ethylmaleimide-sensitive attachment protein receptor complexes, essential for extracellular vesicle release at the presynapse (Miki et al., 2025). Extracellular vesicles contain neurotransmitters in the hippocampus. Therefore, the reduced secretion of extracellular vesicles due to α-synuclein oligomers is highly likely to be the causative event of memory impairment in MSA. The presence of α-synuclein-positive NCIs in the hippocampus of human MSA cases with memory impairment may represent aggregates, wherein α-synuclein oligomers have undergone conversion into aggregates over time. Furthermore, we also observed the same aberrant interactions between α-synuclein oligomers and synaptotagmin 13 in the brain of individuals with dementia with Lewy bodies (Miki et al., 2025), suggesting a shared mechanism of synaptic dysfunction and cognitive dysfunction in synucleinopathies. Recently, Sekiya et al. (2019, 2022) have visualized a widespread distribution of α-synuclein oligomers in the brain of individuals with synucleinopathies. Interestingly, the presence of memory impairment was also associated with the accumulation of α-synuclein oligomers in the hippocampus of patients with Parkinson’s disease. They hypothesized that α-synuclein oligomers initially diffuse into neuropil and then accumulate within neurons, leading to the formation of α-synuclein-positive inclusions and, ultimately, neurodegeneration (Sekiya et al., 2022). Thus, the distribution and amount of α-synuclein may serve as a predictor of the severity of neurodegeneration in the affected regions. However, there are still key questions regarding (1) the precise mechanisms by which the accumulation of α-synuclein oligomers leads to neurodegeneration, and (2) the contributing factors that cause regional burden of α-synuclein oligomers. Further study is warranted to answer these important questions. A schematic representation of the hypothetical mechanisms for memory impairment in MSA is shown in **Figure 1**.

The results in our previous reports (Miki et al., 2020, 2022, 2025) led us to hypothesize that targeting α-synuclein oligomers at the presynapse may be a promising therapeutic strategy for memory impairment in MSA. Tanji et al. (2015) have previously reported that trehalose, which has a 1,1-glycosidic bond with glucose, activates autophagy, one of the intracellular degradation mechanisms. Nevertheless, the oral administration of trehalose did not change the levels of human α-synuclein in the brain of a mouse model of dementia with Lewy bodies (Tanji et al., 2015). This may be because trehalose is broken down into two molecules of glucose in the digestive tract upon oral administration, resulting in incomplete demonstration of its effect in the brain. Alternatively, the amount of trehalose that reached the brain may have been insufficient to elicit a significant effect. Therefore, we tried to deliver trehalose directly to the brain through the nasal cavity (Tanaka et al., 2024). Mass spectrometry confirmed successful delivery and subsequent metabolism of trehalose within the brain of the MSA mouse model. We then performed a behavioral test (object-location memory task) and measured long-term potentiation in the hippocampal neurons. As expected, intranasal administration of trehalose reversed memory in the MSA mouse model to near-normal levels. However, contrary to our expectations, trehalose did not activate autophagy in the brain; instead reduced the amount of α-synuclein oligomer by accelerating the formation of abnormal α-synuclein aggregates (Tanaka et al., 2024). Although abnormal α-synuclein is itself toxic to neurons, multiple studies have reported cytoprotective effects of inclusions by sequestering abnormal α-synuclein from healthy organelles. Therefore, the damage caused to cells by toxic abnormal α-synuclein oligomers was minimized by promoting the formation of aggregates from the toxic abnormal α-synuclein oligomers. These findings led to another hypothesis that inhibition of α-synuclein oligomer formation may also exert favorable effects on memory in the MSA model. To test this hypothesis, we utilized ergothioneine, a substance derived from fungi that possesses potent antioxidant properties (Kimura et al., 2025). We administered ergothioneine nasally to the MSA model to ascertain whether the regulation of abnormal α-synuclein oligomers in the brain would improve memory. Mass spectrometry confirmed the delivery of ergothioneine to the brain. Furthermore, the intranasal administration of ergothioneine was found to inhibit the formation of abnormal α-synuclein oligomers and to improve memory in the MSA model (Kimura et al., 2025). Thus, these results suggest toxic α-synuclein oligomers as a potential culprit in memory impairment of MSA.

The removal of accumulated abnormal α-synuclein in the brain has been a primary focus in the treatment strategies for synucleinopathies, including MSA. Indeed, in December 2024, Pagano et al. reported that long-term administration of anti-α-synuclein antibodies to patients with early-stage Parkinson’s disease suppressed the progression of Parkinson’s disease. However, it should be noted that antibody therapies are extremely expensive, and their effects are limited to the early stages of the disease. In our studies, we have successfully identified the underlying cause of memory impairment in MSA. Most importantly, we have established a novel foundation for MSA treatment strategies by leveraging affordable and highly safe foods as a treatment modality for MSA. Recently, we have developed “Visible Intrabrain (Vintra) Mouse” (Patent Application No. PA22-23). In this model, intranasally administered carriers, which can contain trehalose or ergothioneine, glow upon reaching the brain. The delivery efficacy to the brain can be measured by quantifying light intensity before and after administration. Although we need multiple steps for the safe and efficient delivery of trehalose and/or ergothioneine to humans, intranasal delivery of these drugs to the brain may become a reality in the near future.

*This work was supported by Hirosaki University Priority Research Grant for Future Innovation (to YM), JSPS KAKENHI Grant Numbers 24K10654 (to YM) and 23K24209 (to KW), and the Collaborative Research Project of the Brain Research Institute, Niigata University (to YM).*
